# P-1563. Unraveling a good paradox - Tertiary center experience with Good syndrome and its infectious complications; Insights from 32 cases identified through 2024

**DOI:** 10.1093/ofid/ofaf695.1743

**Published:** 2026-01-11

**Authors:** Ayesha Samreen, Melissa Kerkelis, Omar M Abu Saleh, Avni Joshi, Maria Mendoza

**Affiliations:** Mayo Clinic, Rochester, MN; Mayo Clinic, Rochester, MN; Mayo Clinic, Rochester, MN; Mayo clinic, Rochester, Minnesota; Mayo Clinic, Rochester, MN

## Abstract

**Background:**

Good syndrome (GS) is an under-recognized adult-onset immunodeficiency characterized by the coexistence of thymoma and hypogammaglobulinemia. It is marked by recurrent infections, combined B- and T-cell immunodeficiency, and frequent autoimmune manifestations. Its rarity, unclear pathogenesis, and often delayed diagnosis contribute to its clinical complexity.

**Figure:**
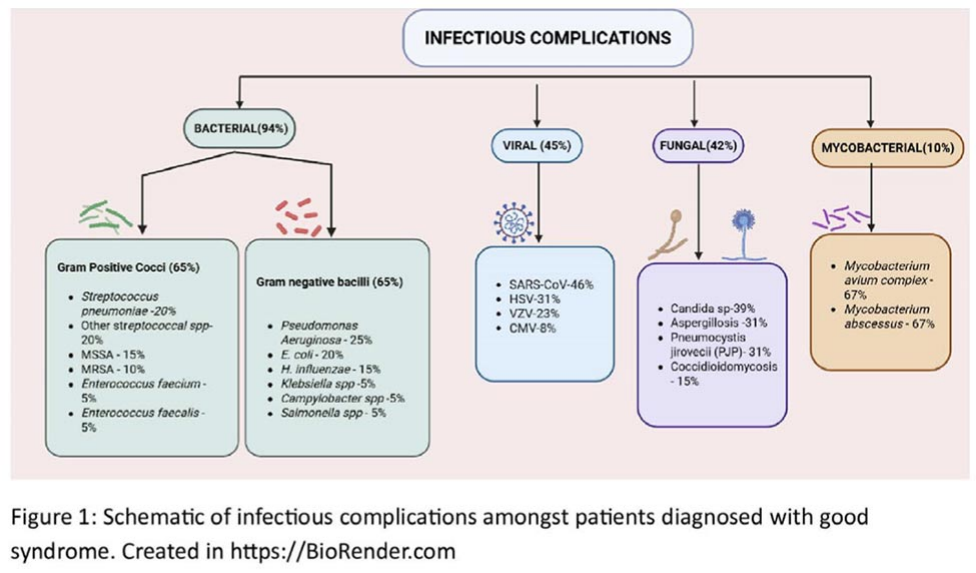
Distribution of Infections by pathogen type in patients with Good Syndrome: the microbiological spectrum

**Figure:**
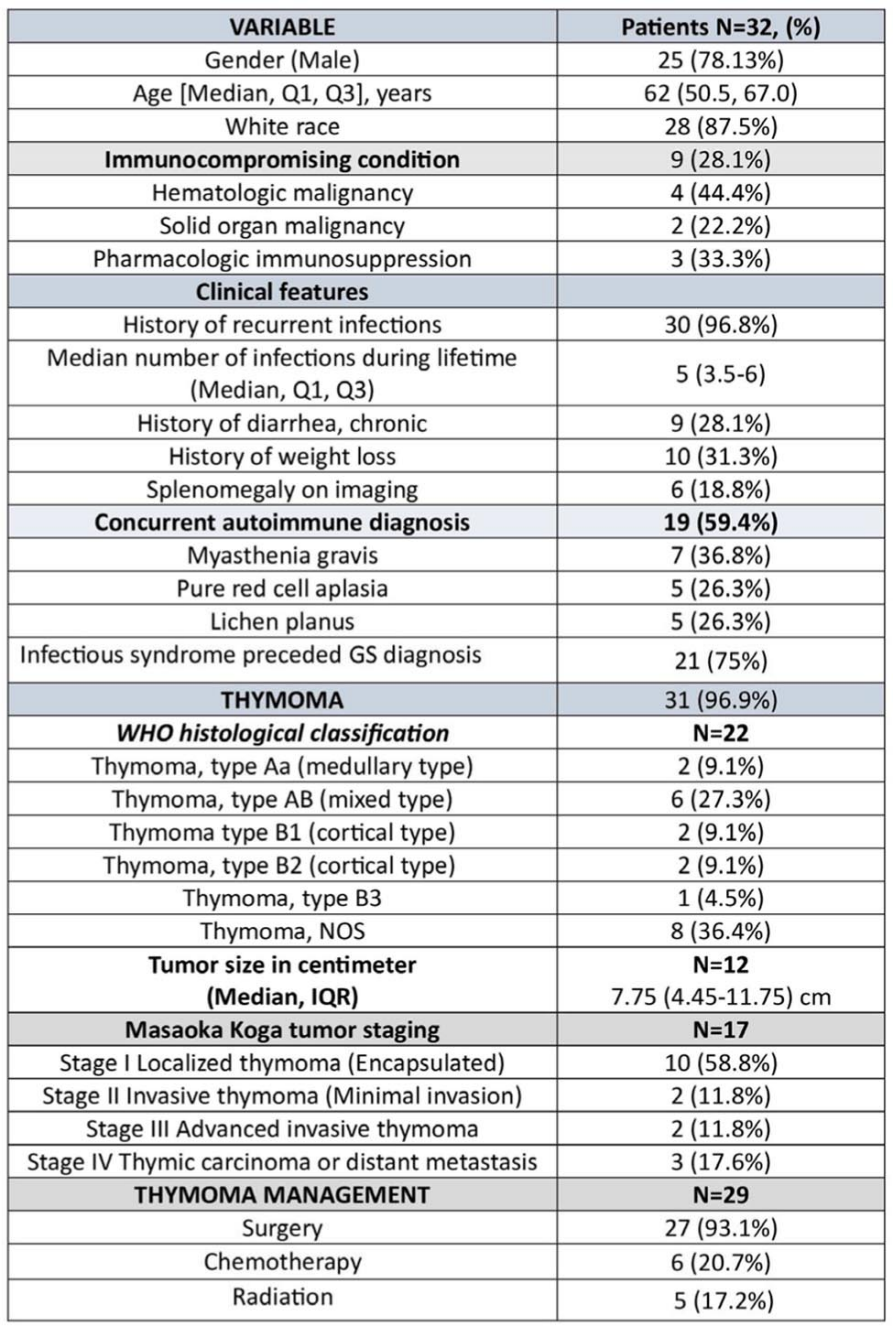
Demographics, clinical characteristics, thymoma classification with staging and management

**Methods:**

We conducted a retrospective review of adults diagnosed with good syndrome at Mayo Clinic up until 2024. We assessed baseline characteristics, clinical features with a particular focus on infectious complications, immunological lab parameters, thymoma histopathology with staging, and management. Descriptive statistics were utilized for analysis.

**Figure:**
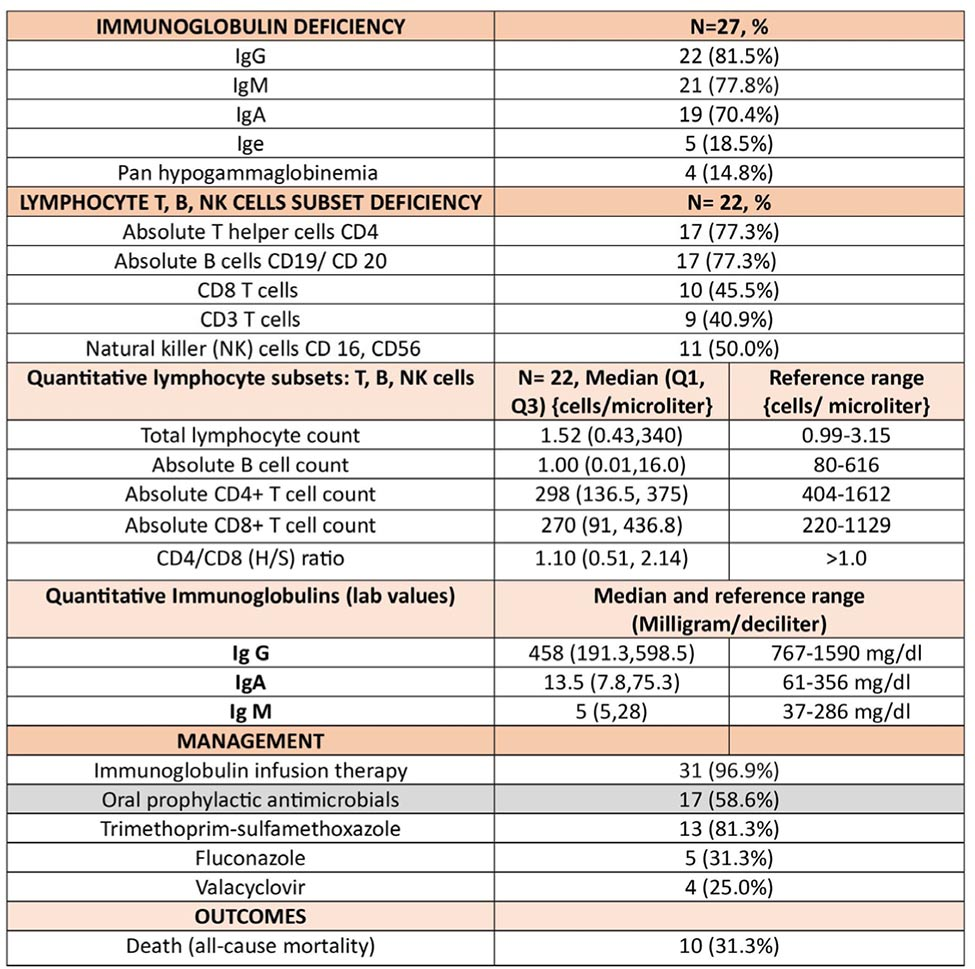
Immunological lab parameters and management of Good syndrome

**Figure:**
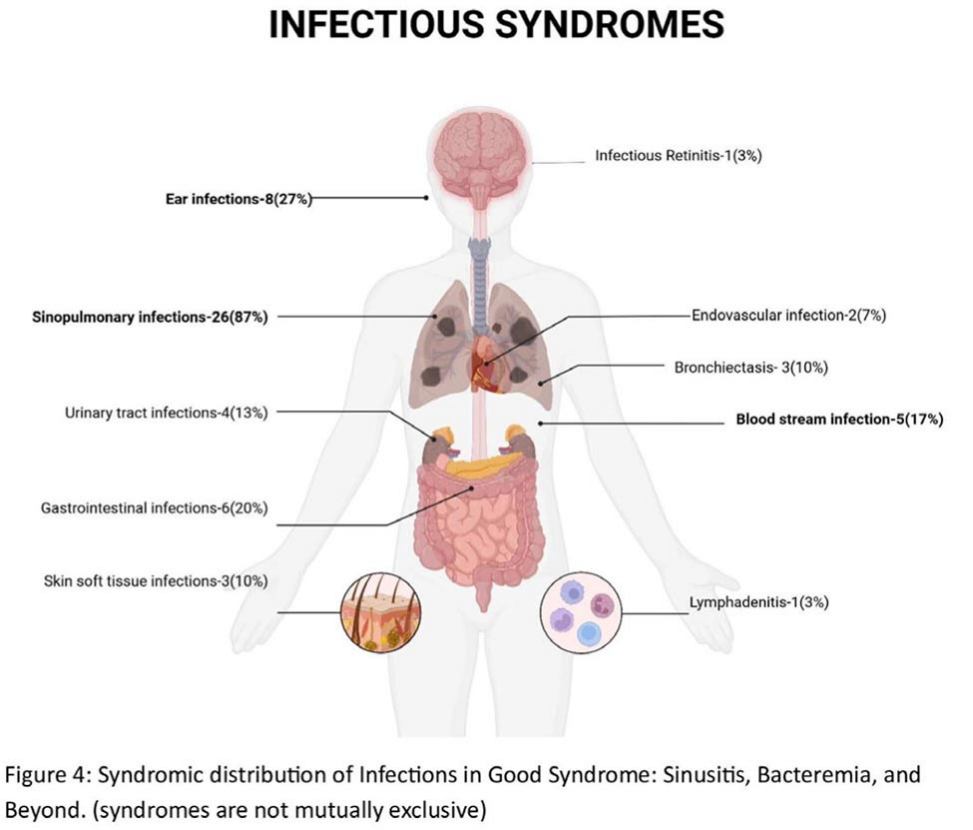

**Results:**

During the study period, Good syndrome was diagnosed in 32 patients; 78% were male, with a median age at diagnosis of 62 years (Table 1). All patients had thymoma, the majority of which were stage I (localized), and most underwent thymectomy as part of management. The median delay in GS diagnosis following thymoma diagnosis was 1,486 days (interquartile range IQR: 406.75–1,992.75).

Infectious complications were observed in 30 of 32 patients (97%), most commonly sinopulmonary infections (87%) (Figure 1). Bacterial infections were documented in 29 patients (94%), viral in 14 (45%), fungal in 13 (42%), and mycobacterial in 3 (10%) (Figure 2).

Immunologic data were available for 22 patients. Reduced CD4+ T-cell counts and severe B-cell lymphopenia were observed in 77%. IgM and IgG deficiencies were the most prevalent, present in 82% of cases. Most patients received monthly immunoglobulin replacement; additionally, 59% were prescribed oral antimicrobial prophylaxis due to their elevated risk of infections.

All-cause mortality was 31% (n=10); among these, 3 deaths (10%) were directly attributable to complications of Good syndrome.

**Conclusion:**

We observed a significant delay (Median of 4.07 years) in the diagnosis of Good syndrome following thymoma detection. Given the high risk of infectious complications and associated mortality, clinicians should maintain a high index of suspicion and consider early immunologic evaluation in adults with recurrent or opportunistic infections, particularly in the context of a known thymoma.

**Disclosures:**

All Authors: No reported disclosures

